# Customized Bone Regeneration—A Retrospective Clinical Follow‐Up Study of the Aesthetic Outcome

**DOI:** 10.1111/clr.14425

**Published:** 2025-03-07

**Authors:** Marcus Seiler, Peer W. Kämmerer, Amely Hartmann

**Affiliations:** ^1^ Clinic for Oral Surgery and Implantology Dr. Seiler Und Kollegen MVZ Filderstadt Germany; ^2^ Department of Oral and Maxillofacial Surgery, Plastic Surgery University Medical Centre of the Johannes Gutenberg University of Mainz Mainz Germany

**Keywords:** aesthetics, bone augmentation procedures, guided bone regeneration, pink aesthetic score, soft tissue

## Abstract

**Objectives:**

This study aimed to evaluate the long‐term stability of soft tissue aesthetics as the primary outcome concerning bone levels (secondary outcome) following customized bone regeneration. Additionally, the influence of flap management techniques on aesthetic outcomes in customized bone augmentation was assessed.

**Material and Methods:**

21 patients (45 implants) who underwent reconstruction of three‐dimensional bone defects using patient‐specific titanium meshes at least 5 years prior were evaluated. The Pink Esthetic Score (PES) was used to assess aesthetic outcomes. Incision lines and flap designs were correlated with aesthetic parameters (primary outcome) and bone levels (secondary outcome). Radiographic measurements of bone levels were taken at implant placement and after more than 5 years. Changes over time in aesthetic outcomes were analyzed using Fisher's Exact Test, Mann–Whitney *U*‐test, and Kruskal–Wallis Test.

**Results:**

Crestal incisions without vertical releasing incisions showed significantly superior outcomes in aesthetics (primary outcome) and bone level mesially (*p* < 0.001) and distally (*p* = 0.001) compared to Poncho flaps or crestal incisions with vertical releasing incisions after > 5 years (secondary outcome). Across parameters, 52.6% to 69.2% of cases achieved a maximum aesthetic rating (“2”). The mean PES score was 11.09 (±3.70) across all implants. The implant survival rate was 100%.

**Conclusions:**

A crestal incision line without releasing incisions was superior in preserving bone levels and achieving favorable aesthetic outcomes. The findings highlight the importance of incision and flap design in the long‐term outcomes of customized bone regeneration. The 100% implant survival rate and lack of side effects further underscore the reliability of this approach.

## 1 Introduction

Guided bone regeneration (GBR) is a well‐established and thoroughly documented technique for augmenting alveolar bone defects before implant placement (Boyne 1969; Dahlin et al. 1988; Kim et al. 2023; Mizraji et al. 2023). In complex three‐dimensional defects, additional mechanical stabilization and space‐maintaining devices are fundamental for achieving predictable outcomes (Kammerer and Al‐Nawas 2023; Troeltzsch et al. 2016). Customized Bone Regeneration (CBR) using individualized titanium meshes was developed to adhere to the principles of GBR, allowing precise reconstruction of extensive and irregular bone defects (Chiapasco et al. 2021; Hartmann et al. 2019; Sagheb et al. 2017; Seiler et al. 2018). Although individualized titanium meshes provide mechanical stability, long‐term aesthetic and structural outcomes data remain scarce (Hartmann et al. 2022).

Soft tissue quality and fixed marginal mucosa are key to long‐term stability in implant‐supported restorations (Monje et al. 2023; Thoma et al. 2018). Even when adequate osseous volume is achieved, ensuring sufficient soft tissue coverage remains a significant challenge, especially in complex three‐dimensional defects. Proper flap management is, therefore, both inevitable and essential for achieving successful outcomes (Fickl et al. 2021). Procedures involving grafts that extend beyond the bony envelope and soft tissue contours are particularly prone to complications, as they present greater challenges in soft tissue coverage. Factors such as scarring, reduced vascularization, and unfavorable flap designs increase the risk of graft exposure (Cucchi et al. 2021; Gu et al. 2022). Additionally, mechanical loss of the graft, bacterial penetration, and food impaction in the augmented area can further contribute to complications such as infection (Nisyrios et al. 2020).

Despite these challenges, long‐term studies on the reliability of customized titanium meshes have demonstrated their potential as an effective bone augmentation technique (Hartmann et al. 2022). However, the extent of mesh exposure during the healing process and the presence or absence of infection remain critical factors influencing outcomes. Importantly, there is a lack of studies assessing long‐term aesthetic results, crucial for patient satisfaction and overall clinical success.

To the best of our knowledge, this is the first study to present long‐term aesthetic outcomes of the CBR technique (primary outcome). We aim to provide insights into optimizing CBR protocols to achieve stable and predictable results by addressing this gap. Accordingly, this study aims to evaluate the long‐term aesthetic stability of the CBR technique, with a specific focus on the interplay between flap management and aesthetic outcomes.

## 2 Materials and Methods

This retrospective clinical follow‐up study followed the ethical principles outlined in the Declaration of Helsinki (World Medical Association 2013). Ethical approval was obtained from the local ethics committee of Baden‐Württemberg (F‐2022‐019‐z). Written informed consent was obtained from each participant before their inclusion in the study. The aesthetic parameters of the PES score were defined as the primary outcome. Marginal bone level mesial/distal (MBL m/d) (after > 5 years) was designated as the secondary outcome. Influence of various parameters on these factors was evaluated. This study was following the appropriate guidelines and strobe checklist.

### 2.1 Case Selection

**FIGURE 1:**
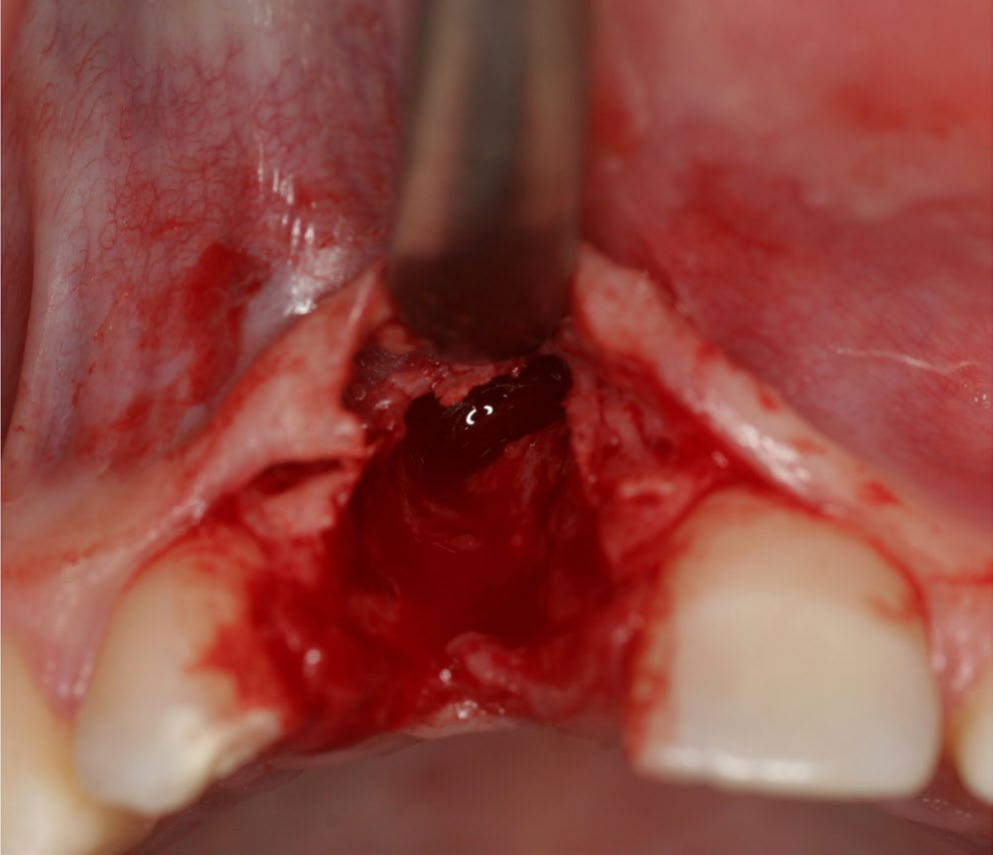
Complex defect situation.

**FIGURE 2:**
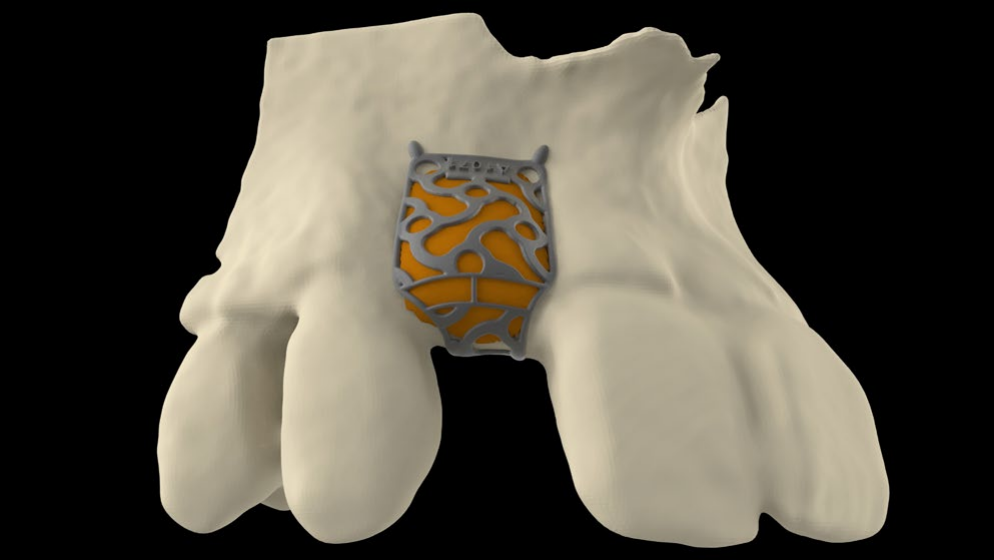
Digitally added grafting volume (orange) and titanium mesh on the bony defect.

**FIGURE 3:**
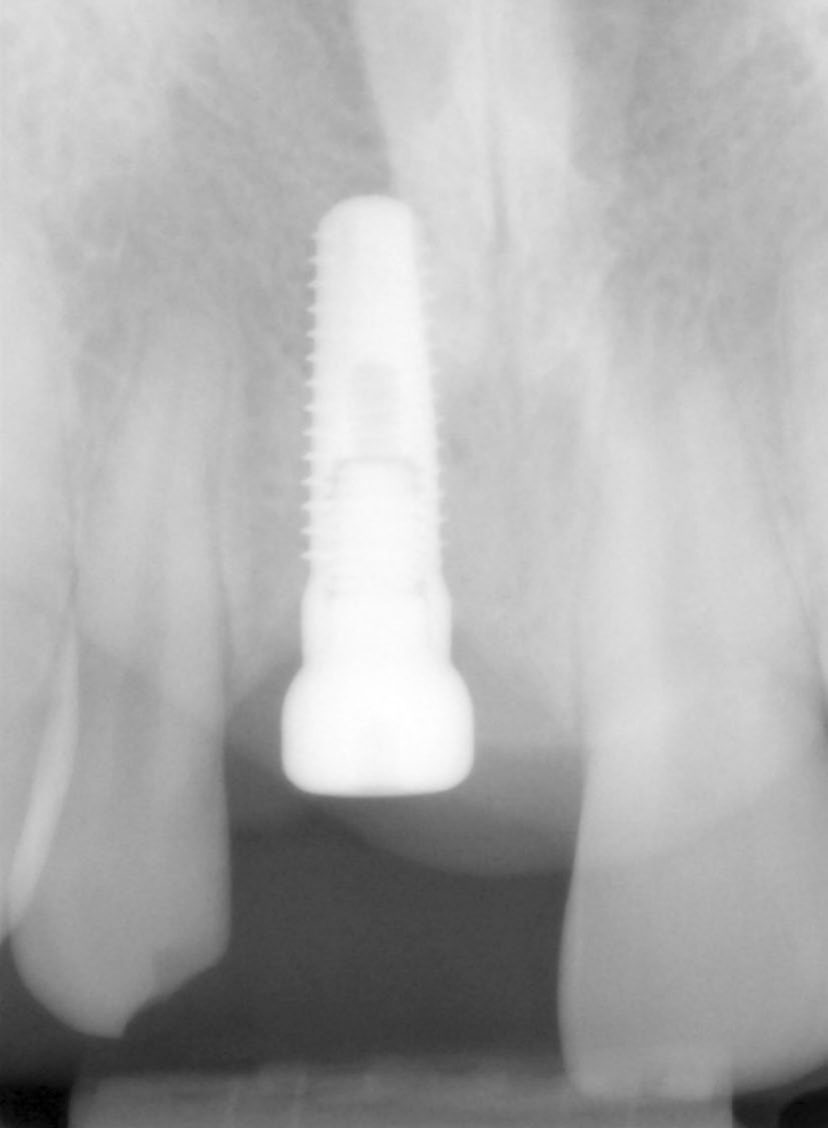
Uncovering the implant in 2016.

**FIGURE 4:**
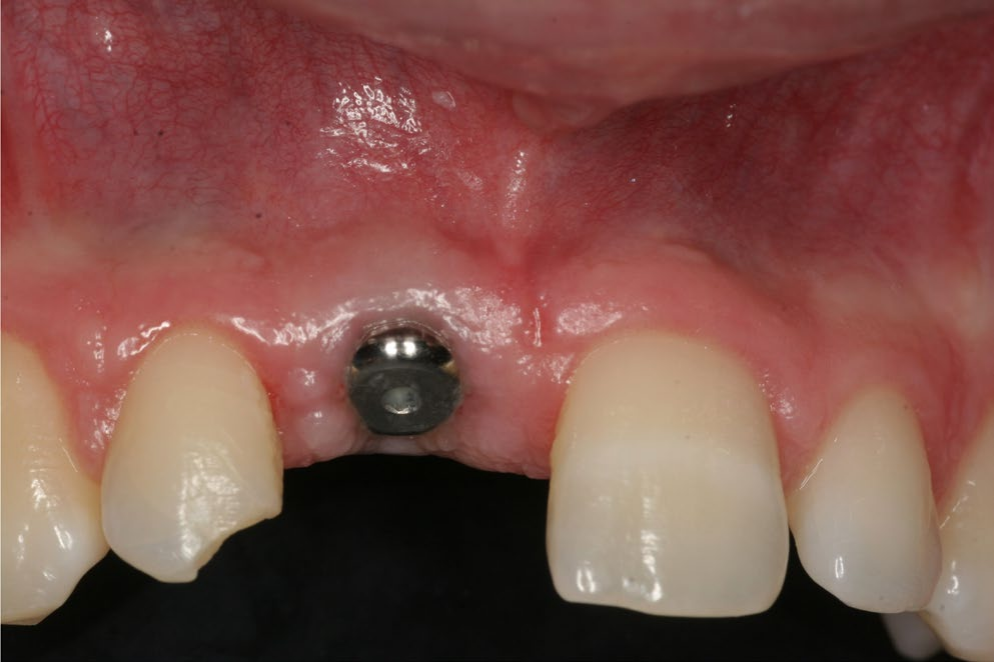
Soft tissue situation at the time of uncovering.

**FIGURE 5:**
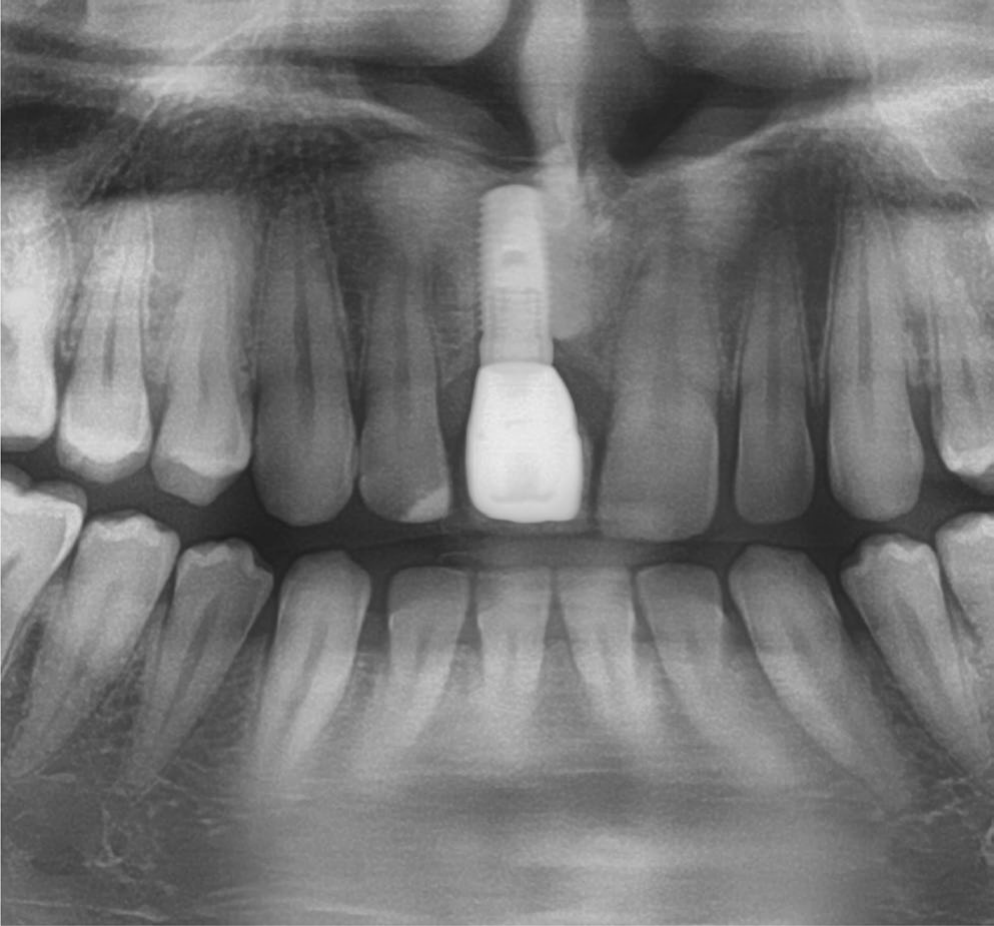
X‐Ray taken at the follow‐up appointment in 2022.

Patients with a complex general medical history, such as chronic pain conditions, congenital diseases, neurological disorders, or malignancies, were excluded from the study. Patients with complex, three‐dimensional alveolar bone defects were treated using a patient‐specific titanium mesh (YxOss CBR, ReOSS, Filderstadt, Germany) in combination with particulate grafting material comprising autogenous bone and xenograft particles (BioOss, 0.25 mm, Geistlich, Wolhusen, Switzerland) in a 1:1 ratio (Figures 1, 2, 3, 4, 5). Patients with small alveolar bone defects (< 3 mm, as defined by Yu et al. (2023)) were excluded from the study. In all cases, tension‐free wound closure was mandatory. This was accomplished using two‐layer suturing: a deep mattress suture with resorbable material, followed by non‐resorbable single stitches for the superficial layer. For 14 days postoperatively, a preformed vacuum‐formed splint was applied to cover the defects to protect the surgical site and facilitate healing. After a healing period of 6–8 months, the individual titanium mesh was removed, and implant placement was performed following standard guidelines. After another healing phase of 3–4 months, the prosthetic oral rehabilitation was completed by the referring dental practice.

The Screw Line implant system (Camlog, Wimsheim, Germany) was used in 44 cases, while the Astra Tech Implant System (Dentsply Sirona, Bensheim, Germany) was utilized in one case. Of the augmentation procedures and implant placements, 57.9% were performed in the upper jaw and 42.1% in the lower jaw. Regarding the sextants and location of the implants, in S1 (17–14, *n* = 5 implants, 11%), in S2 (13–23, *n* = 5 implants, 11%), in S3 (24–27, *n* = 16 implants, 35.2%), in S4 (37–34, *n* = 7 implants, 15.4%), in S5 (33–43, *n* = 1 implant, 2.2%), in S6 (44–47, *n* = 11 implants, 24.2%) where placed, S2, 13–23 In 20 patients, one implant was placed. Six Patients underwent surgery with two adjacent implants and five patients with three implants. Each implant (and its soft tissue situation) was evaluated independently as presented in Furhauser et al. (2005). The situation was compared to the adjacent teeth or contralateral tooth if the adjacent tooth was an implant. Each papilla (m/d) was individually assessed dependent on the respective implant.

### 2.2 Measurements

#### 2.2.1 Primary Outcome

##### 2.2.1.1 Flap Management and Incision Techniques

**FIGURE 6:**
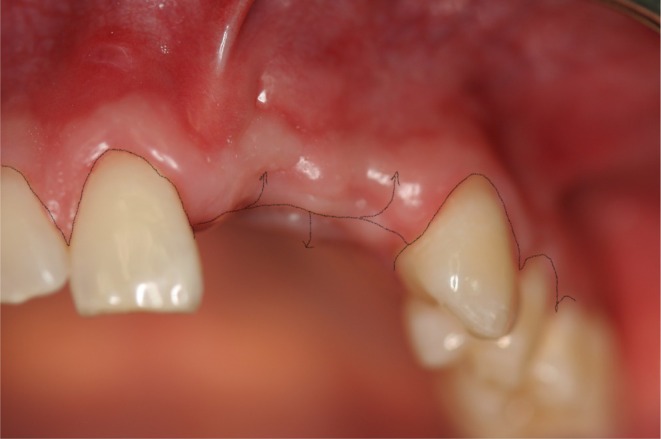
Schematic scenario of a flap management without releasing incision (part 1).

**FIGURE 7:**
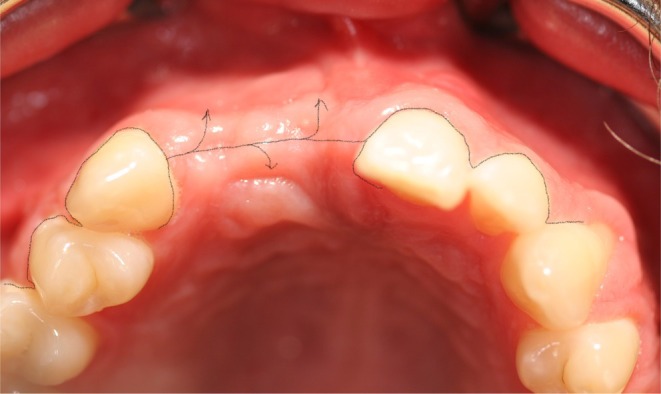
Schematic scenario of a flap management without releasing incision (part 2).

**FIGURE 8:**
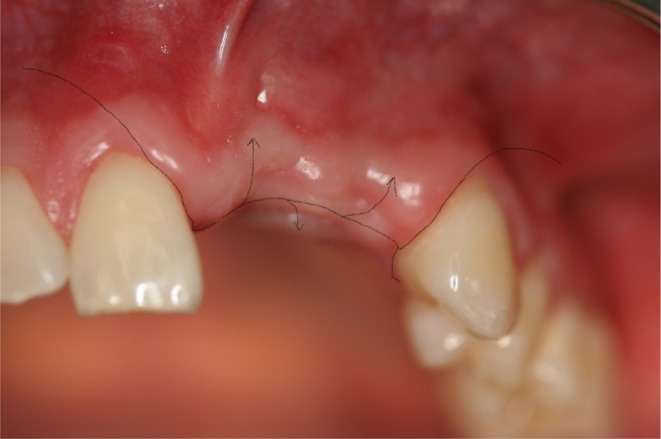
Schematic scenario of a flap management with releasing incision (part 1).

**FIGURE 9:**
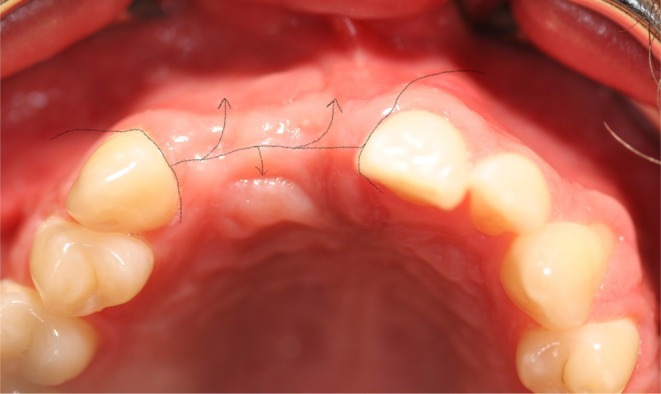
Schematic scenario of a flap management with releasing incision (part 2).

**FIGURE 10:**
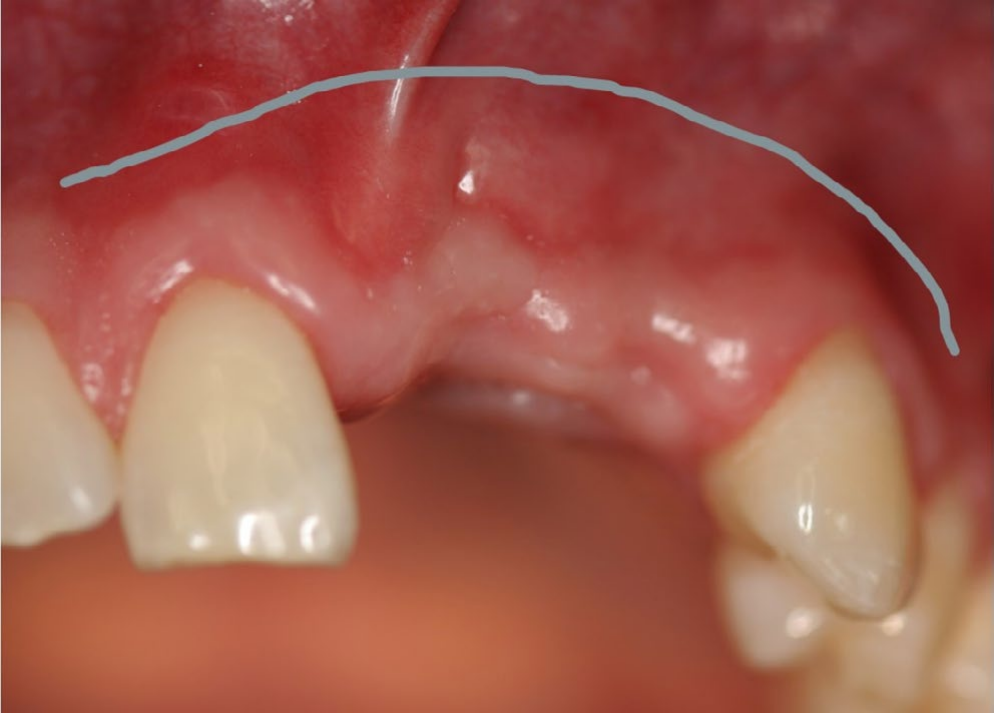
Schematic scenario of a flap management with poncho flap (part 1).

**FIGURE 11:**
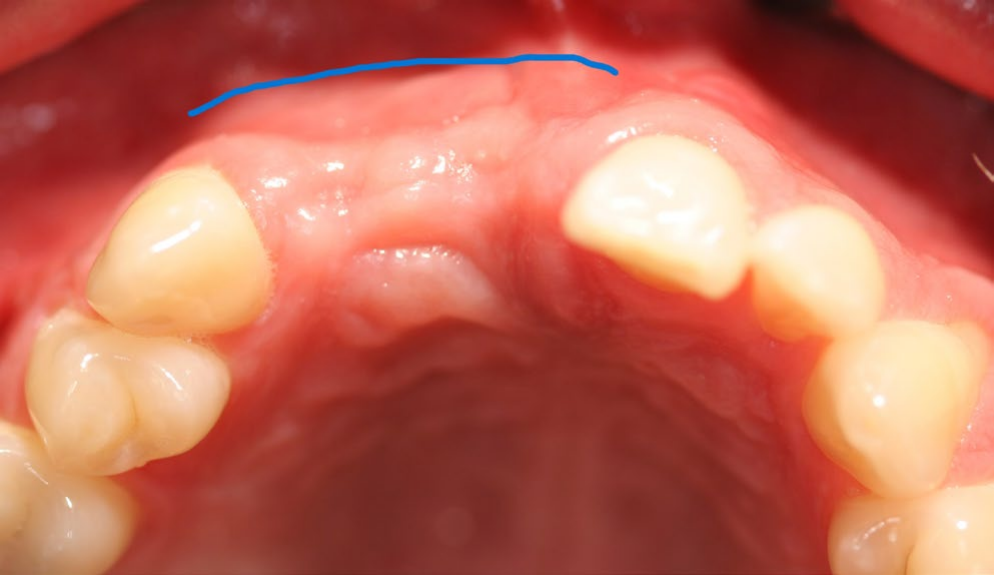
Schematic scenario of a flap management with poncho flap (part 2).

The techniques were categorized into three groups: crestal incision without a releasing incision (extending to a minimum of two neighboring teeth) (Group A, *n* = 21, Figures 6 and 7), crestal incision with vertical releasing incision (Group B, *n* = 10, Figures 8 and 9), and the poncho technique (Group C, *n* = 14, Figures 10 and 11). For the poncho technique, the incision was located buccally from the alveolar crest so that minor wound dehiscences would not lead to direct contact with the graft (Kammerer and Al‐Nawas 2023; S. Wang and De Santis 2024).

**FIGURE 12:**
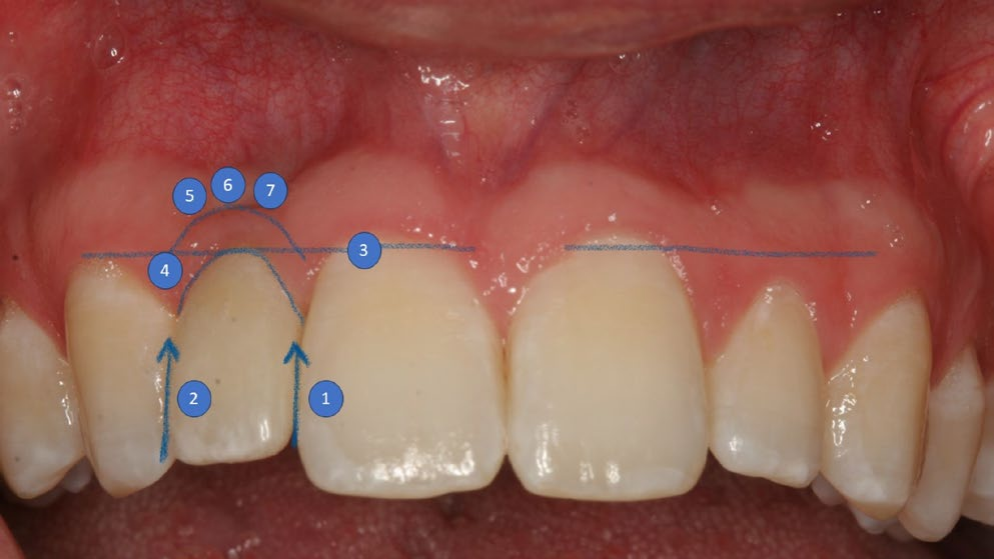
Pink Esthetic Score with the seven parameters (adopted from Furhauser et al. (2005)). Clinical picture of a situation 5 years after complex bone augmentation procedure. (1) Mesial papilla. (2) Distal papilla. (3) Level of soft tissue margin. (4) Soft tissue contour. (5) Soft tissue colour. (6) Alveolar process. (7) Soft tissue texture.

**FIGURE 13:**
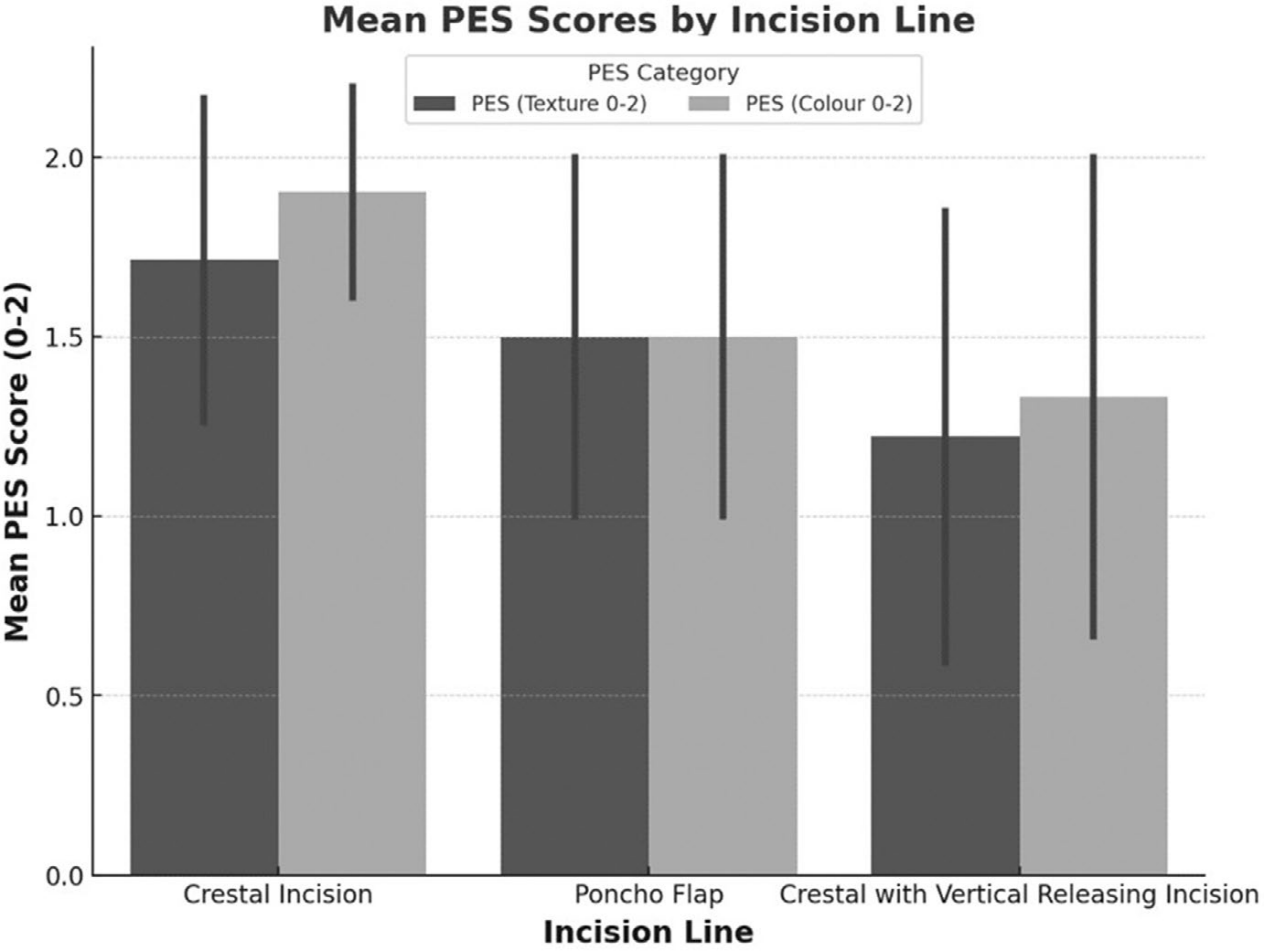
Influence of incision technique and flap design on aesthetic/PES‐parameter color.

**FIGURE 14:**
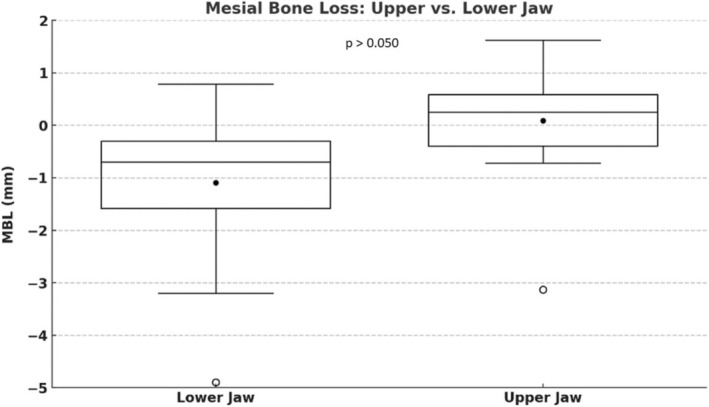
MBL (Marginal bone loss) mesial: upper versus lower jaw.

**FIGURE 15:**
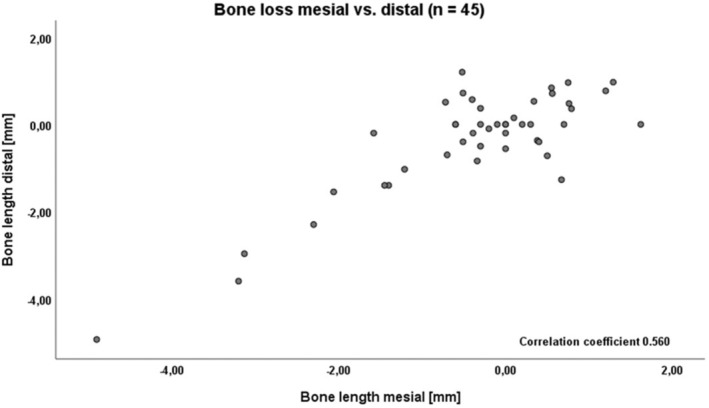
Bone level relation (mesial and distal after 5 years, spearman correlation 0.560).

After over 5 years, a follow‐up investigation of the grafted sites was conducted as part of routine daily practice:

##### 2.2.1.2 Pink Esthetic Score (PES)

Soft tissue evaluation was performed based on the principles established by Furhauser et al. (2005). The seven parameters documented were papilla mesial and distal, tissue contours, mucosal level, alveolar process, coloring, and texture (Figure 12). These parameters were compared to a reference tooth, with patients required to have adjacent teeth and/or a contralateral side to provide a basis for documenting the soft tissue situation and serving as a reference.

Each parameter was graded on a scale of “0,” “1,” and “2,” where “0” indicated a complete loss of aesthetic appearance, and “2” represented the ideal version. Specifically:

- Papilla mesial and distal: “0” indicated missing or absent papilla, “1” represented incomplete papilla, and “2” indicated complete papilla.
- Soft tissue contours: “0” was recorded for unnatural contours, “1” for virtually natural contours, and “2” for fully natural contours.
- Gingival level: A discrepancy > 2 mm compared to the reference tooth was rated as “0” (major discrepancy), 1–2 mm as “1” (minor discrepancy), and < 1 mm as “2” (no discrepancy).
- Alveolar process: “0” described an obviously resorbed process, “1” a slightly resorbed process, and “2” no resorption.
- Coloring and texture: “0” indicated a clear and obvious difference, “1” a slight to moderate difference, and “2” no difference.

#### 2.2.2 Secondary Outcome

##### 2.2.2.1 Marginal Bone Level Mesial/Distal (MBL m/d)

Influence of various parameters (incision technique, exposure rate, Periodontal Phenotype, clinical parameters and implant stability) on bone levels was assessed. Implant survival was evaluated.

##### 2.2.2.2 Exposure Rate

The exposure rate was evaluated following the methodology described by Hartmann et al. (2019):

A: punctual exposure of the mesh.

B: exposure has the size of one tooth width.

C: complete exposure of the mesh.

D: No exposure.

##### 2.2.2.3 Periodontal Phenotype

The periodontal phenotype was documented according to Muller et al. (2000):

- Cluster A1 and A2 (thin gingiva type, slender tooth shape, differentiated by gingival width)
- Cluster B (thick, wide gingiva, quadratic tooth form)

##### 2.2.2.4 Clinical Parameters

Bleeding on probing (BOP), Suppuration and Pain were documented as “Positive/Negative”.

##### 2.2.2.5 Implant Stability

It was measured using the Periotest device (Periotest M, ImplAG, Lorsch, Germany) (Geurs et al. 2002).

### 2.3 Patients

A total of 21 patients (11 females and 10 males) with 45 implants were included in this follow‐up study. The evaluation was conducted for each implant. As part of a routine clinical examination, these patients were clinically and radiologically evaluated. The mean follow‐up period was 5.69 ± 0.38 years. The mean age of the patients was 66.87 years (±16.69, range: 21–88 years). Female patients had a mean age of 68.25 years (±15.06, range: 42–88 years), while male patients had a mean age of 64.59 years (±19.36, range: 21–86 years). The patient‐specific titanium mesh remained in situ for an average of 5.39 ± 2.23 months.

### 2.4 Radiographic Analyses

The relationship between mesial and distal bone levels and aesthetic outcomes was analyzed as well as the impact of the augmentation region on bone level. Bone level changes (MBL) were assessed by comparing orthopantomograms (OPT) taken at the time of implant placement with those obtained at the follow‐up examination ≥ 5 years. It was measured (by one surgeon A.H., not the one performing the surgical procedure M.S.) by using ImageJ 1.53 k (Schneider et al. 2012), evaluating a difference between the two time points. The top of the inserted implant and the respective bone level mesial and distal post‐surgery was compared to the top of the inserted implant and the bone level mesial and distal at follow‐up ≥ 5 years. Radiographic distortion was excluded according to Gómez‐Román et al. (1995) and Gomez‐Roman and Launer (2016). Implant diameter (as provided by the manufacturer) was set as a reference. It was individually multiplied, defined for each radiograph.

Flap and incision designs were correlated with both aesthetic outcomes and bone levels. Additionally, the potential influence of peri‐implantitis‐related factors and other parameters on bone levels and aesthetic results was evaluated, following the methodology described earlier (Hartmann et al. 2022).

### 2.5 Statistics

Statistical analysis was performed using SPSS statistical software version 26 (SPSS, IBM, Ehningen, Germany). The aesthetic outcomes, including the Pink Esthetic Score (PES), were assessed in relation to various flap and incision designs as well as peri‐implant bone levels. PES was utilized as a standardized measure to evaluate the esthetic integration of implants in soft tissue. Changes in bone levels were quantitatively analyzed by comparing radiographic measurements from orthopantomograms obtained at the time of implant placement with those from follow‐up examinations conducted more than 5 years later.

To explore potential associations between peri‐implantitis‐related factors, surgical techniques (flap and incision designs), bone levels, and aesthetic parameters, statistical tests were performed. Analyses were used to assess the strength and significance of these relationships.

The changes in bone levels were expressed in millimeters, and the distribution of these changes was examined for normality. Depending on the distribution, either parametric tests (e.g., paired *t*‐tests) or nonparametric tests (Wilcoxon test, Kruskal–Wallis) were used to evaluate differences over time. For categorical variables, associations were analyzed using Fisher's exact test, as appropriate. The level of statistical significance was set at *p* < 0.050. Statistical methods and results are visualized in Table 1.

**TABLE 1:** Comprehensive table reporting demographic data, statistical methods, results, and parameters investigated.

Parameter	Summary
*Descriptive data*	
Age (±16.69 SD)	mean: 66.87 min. 21 years, max. 88 years
Sex (f: female, m: male)	f: *n* = 28 (62.22%) m: *n* = 17 (37.78%)
Exposure A: punctual exposure of the mesh B: exposure has the size of one tooth width C: complete exposure of the mesh D: No exposure	A: *n* = 3 (6.67%) B: *n* = 1 (2.22%) C: *n* = 2 (4.44%) D: *n* = 39 (86.67%)
Periodontal type (A1, A2: thin gingiva type, slender tooth shape, differentiated by gingival width, B: thick, wide gingiva, quadratic tooth shape)	A1: *n* = 25 (55.56%) A2: *n* = 4 (8.89%) B: *n* = 16 (35.56%)
Incision line (A: marginal incision, B: marginal incision with vertical relief, C: Poncho flap)	A: *n* = 21 (46.67%) B: *n* = 10 (22.22%) C: *n* = 14 (31.11%)
BOP (0: negative, 1: positive)	0: *n* = 43 (95.56%) 1: *n* = 2 (4.44%)
Suppuration (0: negative, 1: positive)	0: *n* = 44 (97.78%) 1: *n* = 1 (2.22%)
Pain (0: negative, 1: positive)	0: *n* = 44 (97.78%) 1: *n* = 1 (2.22%)
Periotest value (time of uncovering) (±1.64 SD)	mean: −6.46 (min. −8, max. −2)
Periotest value (> 5 years) (±2.39 SD)	mean: −5.33 (min. −8, max. −1) *p* = 0,005*
*Statistical analysis*	
PES (±3.70 SD)	mean: 11.09
Aesthetics (PES)—location of implants (sextants)	n.s.^a^
Aesthetics (PES)—location of implants (upper/lower jaw)	n.s.^a^
Incision line 1: marginal incision/MBL m vs. 0: Poncho flap/MBL m or 2: marginal incision with vertical relief/MBL m	1/MBL m (0.30 ± 0.60 mm) 0/MBL m (−1.23 ± 1.41 mm) 2/MBL m (−0.55 ± 1.31 mm) (*p* < 0.001)**
Incision line 1: marginal incision/MBL d vs. 0: Poncho flap/MBL d or 2: marginal incision with vertical relief/MBL d	1/MBL d (0.27 ± 0.46 mm) 0/MBL d (−1.11 ± 1.52 mm) 2/MBL d (−0.65 ± 1.20 mm) (*p* = 0.001)**
Implant survival rate (0: negative, 1: positive)	1: *n* = 45 (100.00%)
MBL m lower jaw vs. MBL m upper jaw	−0.98 ± 1.42 mm vs. 0.08 ± 0.89 mm *p* = 0.005*
MBL d lower jaw vs. MBL d upper jaw	−0.86 ± 1.48 mm vs. −0.04 ± 0.83 mm *p* = 0.031*
Exposures on MBL m A: punctual exposure of the mesh B: exposure has the size of one tooth width C: complete exposure of the mesh D: No exposure	A–C: mean: −0.37 mm D: mean 0.36 mm *p* = 0.917***
Exposures on MBL d A: punctual exposure of the mesh B: exposure has the size of one tooth width C: complete exposure of the mesh D: No exposure	A–C: mean: −0.39 mm D: 0.39 mm *p* = 0.897***

Abbreviations: BOP, bleeding on probing; d, distal; m, mesial; MBL, marginal bone level; *n*, number; PES, Pink Esthetic Score; SD, standard deviation.

^a^ Fisher's Exact Test.

* Wilcoxon test.

** Kruskal–Wallis Test.

*** *t*‐test.

## 3 Results

### 3.1 Primary Outcome

#### 3.1.1 Flap Management and Incision Techniques

Regarding aesthetics indicated by the PES parameters and former flap design, the crestal incision without vertical releasing incision showed significantly better results for the “color” parameter (*p* = 0.004). Maximum “2” scores for “color” were achieved in 90.5% (*n* = 19/21) of cases with crestal incisions, compared to 50% (*n* = 7/14) for the Poncho flap technique and 40% (*n* = 4/10) for crestal incision with vertical releasing incision. A similar trend was observed for other PES parameters (Figure 13).

#### 3.1.2 Pink Esthetic Score (PES)

The mean PES was 11.09 (±3.70 SD) across 45 implants. A maximum score of “2” was achieved in 53.3% of cases (*n* = 24) for both mesial and distal papilla height. Similarly, “2” was scored in 55.6% of cases (*n* = 25) for tissue contours, gingival level, and texture; 62.2% (*n* = 28) for the alveolar process; and 66.7% (*n* = 30) for coloring. One case exhibited unsatisfactory results (“0”) for all parameters. Compromised results were observed in three cases for distal papilla height and alveolar process and in two cases for mesial papilla height.

When comparing the upper and lower jaws, no significant differences in the seven aesthetic parameters were observed. Similar nonsignificant differences were evaluated when comparing the sextants (17–14, 13–23, 24–27, 47–44, 43–33, and 34–37).

### 3.2 Secondary Outcome

#### 3.2.1 Marginal Bone Level Mesial/Distal (MBL m/d)

Crestal incision without vertical releasing incision resulted in significantly better mesial bone levels (0.30 ± 0.60 mm) compared to crestal incision with vertical releasing incision (−0.55 ± 1.31 mm) and the Poncho flap technique (−1.23 ± 1.41 mm) (*p* < 0.001).

For distal bone levels, similar superiority (*p* = 0.001) was noted with crestal incision without vertical releasing incision (0.27 ± 0.46 mm) compared to crestal incision with vertical releasing incision (−0.65 ± 1.20 mm) and Poncho flap (−1.11 ± 1.52 mm).

The implant survival rate was 100%. The lower jaw exhibited significantly more bone loss compared to the upper jaw. Mesial bone loss was −0.98 ± 1.42 mm in the lower jaw versus 0.078 ± 0.89 mm in the upper jaw (*p* = 0.005) (Figures 14 and 15). Distal bone loss was −0.86 ± 1.48 mm in the lower jaw versus −0.037 ± 0.83 mm in the upper jaw (*p* = 0.031).

#### 3.2.2 Exposure Rate

Exposure rates were documented as follows:

- Group A: 7% (*n* = 3),
- Group B: 2% (*n* = 1),
- Group C: 4% (*n* = 2),
- Group D (uneventful healing): 87% (*n* = 36).

Exposure (classified as A–C) had no significant impact on mesial bone levels (mean: −0.37 mm for A–C vs. −0.36 mm for D, *p* = 0.917) or distal bone levels (mean: −0.39 mm for A–C vs. −0.39 mm for D, *p* = 0.897). PES scores were impaired in cases with exposure, regardless of the exposure classification (A, B, or C).

#### 3.2.3 Periodontal Phenotype

The periodontal phenotype (Muller et al. 2000) was categorized as:

- A1: 55.6% (*n* = 25),
- A2: 8.9% (*n* = 4), both representing the “thin phenotype” (64.5%, *n* = 29).
- B: 35.5% (*n* = 16), representing the “thick phenotype.”

The thin phenotype (A1/A2) achieved higher PES scores for mesial and distal papilla compared to the thick phenotype (B). Severe exposures were more common with the thick phenotype, occurring in 12.5% of cases.

#### 3.2.4 Clinical Parameters

Bleeding on probing (BOP) was positive in 4.4% of cases (*n* = 2). One patient (*n* = 1) reported occasional pain but did not complete a pain questionnaire. Suppuration and radiopacity were documented in one case each, with no overlap.

#### 3.2.5 Implant Stability

At the time of implant uncovering, the mean Periotest value was −6.46 ± 1.64 (min. −8, max. −2). After more than 5 years, the mean value was −5.33 ± 2.39 (min. −8, max. −1). Stable values (> 0) were recorded in 93.3% of implants, with a maximum score of “8” in 26.7%. A reduced value was observed in 37.8% of implants, while 13.3% showed improvement. In 8.9% of cases, Periotest values remained unchanged. In 40% of cases, comparisons were not possible due to incomplete data.

## 4 Discussion

Customized titanium meshes aim to reconstruct complex alveolar bone defects (Chiapasco et al. 2021; Cucchi et al. 2021; Sagheb et al. 2017). Long‐term results are still rare (Hartmann et al. 2022). In general, the long‐term success of an implant is based on sufficient hard tissue around the implant, implant placement according to the prosthetic backward planning, and stable soft tissue (Buser et al. 2004).

100% implant survival was reported for this study compared to 88%–97% (Hartmann et al. 2022; Nguyen et al. 2018; Shi et al. 2016; Singh et al. 2019) in other studies with various grafting procedures. Describing an implant survival rate does not include the presentation of an aesthetic outcome that represents a real long‐term success for the patient. Therefore, in this study, the aesthetic parameters were added and analyzed as the primary outcome. As a diagnostic tool, the PES was used, which is a well‐established tool to describe soft tissue situation around implants (Furhauser et al. 2005; Wittneben et al. 2023). The mean PES score was 11.09 (±3.70) compared to a maximum achievable score of 14. This indicates a successful aesthetic outcome for cases with former complex defects. Scars from former surgeries might also have impaired the outcome.

No differences were documented between the upper and lower jaw and between the sextants concerning all PES parameters. The high score value was achieved in 53.3%–66.7%. This excludes any side or individual effects and may be related to the reliability of the technique itself. It is necessary to consider that the number of cases is relatively small, and further studies with a larger patient sample are needed. One might also criticize the PES for being established only in single‐tooth implant crowns compared to a natural reference tooth. Because of this, any implant‐supported crown was compared to another reference tooth (contralateral). In the literature, it was described as an objective way of reporting soft tissue situations in grafting procedures (Chen et al. 2023; Li et al. 2021). In the original publication, the photographs were assessed by various observers (orthodontists, surgeons, prosthodontists, and dental students) (Furhauser et al. 2005). In this study, the follow‐up appointment was undertaken by another surgeon (A.H.) with expertise in grafting procedures to exclude any bias by performing the follow‐up by the same surgeon (single arm) who had done the grafting procedures (M.S.).

A proper flap preparation and incision technique is crucial in grafting procedures. This study showed for the first time, together with customized bone regeneration, the superiority of flap management. One might assume that the avoidance of a vertical releasing incision and strictly preparing a full‐thickness flap with an extension to the adjacent teeth (minimum 2) brought a significant benefit for bone level after 5 years and for aesthetics. Especially, the color of the soft tissue was significantly improved compared to the other techniques. This may be due to the avoidance of scars, no additional lateral movement of the flap, and persistent soft tissue nutrition. These findings are in accordance with the biological principles for irritation‐free wound healing and thus predictable bone regeneration according to Wang and Boyapati (2006) using the “PASS” criteria. These can be applied to all grafting procedures. They include sufficient primary wound closure (“P”), sufficient angiogenesis (“A”) to provide the necessary blood supply and undifferentiated mesenchymal cells, then space maintenance/creation (“S”) to provide sufficient space for ingrowth bone, and finally mechanical stability (“S”) of wound and implant. A wrong treatment may lead to exposures of the graft and, in cases with titanium meshes, to a partial loss of the graft (Cunha et al. 2022; Hartmann and Seiler 2020). According to the present findings, they are also related to aesthetics.

Besides a missing long‐term aesthetic outcome, inappropriate soft tissue management may lead to exposures of the graft and titanium mesh during the healing period. Considering patient‐specific titanium meshes, 25% of exposures were described in the literature (Gu et al. 2022; Poli et al. 2022). In this study, 13% of the exposures occurred, representing an average rate compared to the literature (Chiapasco et al. 2021; Poli et al. 2022; Xie et al. 2020). As indicated in a previous study by Hartmann et al. (2022), exposures did not influence bone level after > 5 years. It was described that exposure to the titanium mesh during the healing period did not influence the implant survival rate. The same applies to this study for long‐term aesthetics.

The association between the appearance of severe exposures in cases with a thick phenotype may be due to the previous surgeries in the complex cases of the study. Scars and reduced vascularization may trigger the occurrence of exposures independent of periodontal phenotype. This contradicts Maroso et al., who evaluated a correlation between peri‐implant mucosal thickness and recessions (Maroso et al. 2015). This was supported by Yuan et al. (2020), who described a relationship between peri‐implant mucosal thickness and bone thickness, indicating aesthetic results. In patients with free gingival graft surgery (Karakis Akcan et al. 2019), clinical parameters showed no statistically significant differences when comparing thick/thin phenotype.

In any flap preparation and incision technique, a consideration of the anatomic principles, according to Kleinheinz, should be considered (Kleinheinz, Buchter, Fillies, et al. 2005; Kleinheinz, Buchter, Kruse‐Losler, et al. 2005). This includes observing the vascularization of a flap as dictated by the anatomy. A mid‐crestal incision is recommended, as are crestal incisions around teeth, and to avoid releasing incisions. Bucco‐oral incisions that cross the ridge should be avoided as well. These findings are supported by the data of our study. Releasing incisions brought no benefit in long‐term aesthetics. One may argue that this flap management will go along with a minimized view of the surgical field. Therefore, correct flap management is essential, as is an extension of the flap around the adjacent teeth (minimum 2). Flap preparation and suturing techniques, as described in the literature (De Stavola and Tunkel 2014; Urban et al. 2018) are useful to gain more volume and to cover the osseous graft and the titanium mesh properly. The size of the mesh had no influence on exposure rate, aesthetics, or bone level.

Limitations of the study might be seen in the lack of a control group treating complex defects differently (like GBR with PTFE‐membrane or split‐block technique). It would have been favorable to perform the surgery without blinding of interventions and outcomes. The study does not report if any patients were lost to follow‐up. Of course, not all the patients who underwent surgery a few years ago were willing to participate in the final examination. Although it is a single‐arm study, this study might be interpreted as pivotal. Randomized controlled clinical trials with more patients are needed. Further studies should include patient satisfaction and quality of life assessments (Hartmann et al. 2022). Concerning the diagnostic tools for measurements, the main limitation of the study might be applying a measurement tool that is very subjective. Although it is an established diagnostic tool for aesthetic outcomes, different investigators might judge each situation differently, as shown in the original publication of Furhauser et al. (2005).

A measurement immediately after the surgical procedure would not have been favorable because of missing prosthetic restoration. Prosthetic restoration was performed by the referral private practices. The study's intention was not to show a change in the soft tissue situation over time but to present and evaluate a clinical situation after > 5 years. Individual factors (periodontitis, oral hygiene, smoking, general health issues) might have influenced the results and aesthetic outcome. Due to the limited number of patients enrolled in this study, in some cases, we had to include patients with more than one tooth being restored. This might have caused irregularities in voting, especially at the adjacent papilla height. Compared to the contralateral tooth, the risk of underestimating the result was reduced. In these cases, with multiple missing teeth, complex defects, and soft tissue situations, the challenges were even more difficult. This situation was more prone to underestimation of the aesthetic results.

The implants were placed according to the guidelines of the respective implant system. The intention was to place the implants at bone crest height or slightly supracrestal to create a prosthetic platform to provide lower stress to peri‐implant tissue (Rito‐Macedo et al. 2021). This was embedded in the backward planning of the customized titanium mesh at the beginning. An immediate connection of the prosthetic abutment would not have positively influenced the bone level and consequently the aesthetic outcome.

### 4.1 Conclusion

This study evaluated a mean PES score of 11.09 (±3.70) compared to a maximum achievable score of 14, indicating a good aesthetic result for the restoration of complex defects. A crestal incision without a releasing incision delivers the best aesthetic results (primary outcome) and presents stable bone volume in the long‐term (primary outcome). This was achieved by tension‐free mobilization of the soft tissue and proper flap extension around the neighboring tooth. The technique itself seems to be reliable in long‐term aesthetics and bone level stability. Limitations of the study might be considered in the lack of a control group and consecutive blinding of the interventions and outcomes. Aesthetic outcomes and bone level stability should be compared to other bone augmentation techniques. Randomized controlled trials with more patients are required.

## Author Contributions

**Marcus Seiler:** conceptualization, methodology, software, data curation, supervision, resources, writing – review and editing. **Peer W. Kämmerer:** writing – review and editing, visualization, formal analysis, data curation, supervision. **Amely Hartmann:** writing – review and editing, writing – original draft, investigation, conceptualization, methodology, validation, formal analysis, project administration, resources, visualization.

## Conflicts of Interest

M. Seiler is the inventor of the customized bone regeneration technique.

## Data Availability

The data that support the findings of this study are available on request from the corresponding author. The data are not publicly available due to privacy or ethical restrictions.
